# Histological and hemodynamic characterization of corpus luteum throughout the luteal phase in pregnant and non-pregnant buffalos in relation to nitric oxide levels based on its anatomical determination

**DOI:** 10.3389/fvets.2022.896581

**Published:** 2022-07-28

**Authors:** Samer M. Daghash, Noha A. E. Yasin, Elshymaa A. Abdelnaby, Ibrahim A. Emam, Ayman Tolba, Yara S. Abouelela

**Affiliations:** ^1^Anatomy and Embryology Department, Faculty of Veterinary Medicine, Cairo University, Giza, Egypt; ^2^Cytology and Histology Department, Faculty of Veterinary Medicine, Cairo University, Giza, Egypt; ^3^Theriogenology Department, Faculty of Veterinary Medicine, Cairo University, Giza, Egypt; ^4^Department of Surgery, Anaesthesiology and Radiology, Faculty of Veterinary Medicine, Cairo University, Giza, Egypt

**Keywords:** buffalos, corpus luteum, Doppler, histology, pregnant

## Abstract

This study aims to compare the complete growth and development of corpus luteum (CL) in domestic buffalos from day 5 until day 40 after ovulation either in pregnant or non-pregnant animals and whether luteal vascularity (LV) with progesterone (P4) and nitric oxide (NO) could determine luteal functionality or not. Pluriparous buffalos (*Bubalus bubalis*) were categorized as pregnant (*n* = 6) or non-pregnant (*n* = 9) after pregnancy check at day 25. Animals were subjected to ultrasound analysis to determine the CL area (cm^2^) and LV. Blood sampling was performed following the Doppler examination. Ovarian tissue samples from non-pregnant buffalo genitalia (*n* = 18) and early pregnant buffalo genitalia (*n* = 3) were collected from great abattoirs. Luteal Doppler indices were lower in the pregnant group, while peak systolic velocity (PSV) was increased (*p* < 0.05) in the same pregnant females. Both P4 and NOMs were elevated (*p* < 0.05) in the pregnant group. There was a positive correlation (*p* < 0.01) between P4 and CL PSV. Based on our macroscopical examination, the CL of non-pregnant buffalos was classified into four stages. Histologically, stage I showed that CL was covered by a highly vascularized connective tissue (CT) capsule. It consisted of small and large lutein cells, whereas stage II was similar to stage I except for the presence of numerous fibroblast cells and vacuolated cells. Stage III was characterized by increasing the number of collagen fibers and the thickness of the blood vessels. Stage IV revealed thickening of the CT capsule and septae, regressed capillaries and arterioles, in addition to shrunken degenerated lutein cells. CL of pregnant buffalos revealed the same structure as CL at stage II. CL area was increased in the pregnant group. The collective data suggested that evaluation of the luteal artery could be extremely helpful to determine the potential benefits of colored and pulsed Doppler in CL vascularization assessment in both luteal and early pregnancy phases.

## Introduction

Buffalos (*Bubalus bubalis*) are a common species in the Middle East (1) and Africa (2), and have great potential due to their critical role in farming and agriculture in those developing countries (3–5). One of the major restraints in the utilization of domestic buffalo reproductive capacity as compared to cattle has been its characteristically poorer reproductive functionality and efficiency (6) that is associated with silent heat, missing many behavioral signs, and lesser conception rates (7, 8). So, studying a basic reproductive pattern is of great importance for enhancing the reproductive efficiency of buffalos (9). The corpus luteum (CL) is the primary reproductive gland responsible for progesterone production, and is required for the establishment and progression of the gestation period; moreover, CL plays a critical role in many reproductive processes, such as successive implantation and embryonic development (10, 11). Histologically, CL consists of cells with a steroidogenic and non-steroidogenic nature (12). The steroidogenic cells, which are responsible for progesterone production, are composed of luteal cells (large and small cells that originate from both granulosa and theca cells), while the non-steroidogenic cells are composed of fibroblasts, endothelial cells, and macrophage (13, 14). Although luteal functions have been evaluated in many studies (15, 16), many specific regulatory factors related to luteal vascularization need future studies, especially in buffalos. The size of the CL at the mid-luteal phase of the estrous cycle ranged from 1 to 2 cm compared to that at pregnancy which ranged from 2 to 2.5 cm (17). Generally, the CL in buffalo is smaller compared to that in cattle (18) and associated with lower progesterone levels, which in turn may affect embryonic mortality (19, 20) and adversely impact the maintenance of pregnancy (21, 22). Some studies have reported that the adequate development of the CL in association with progesterone levels (P4) is needed to prevent embryonic mortality (23); in addition, pregnant buffalos reported a greater diameter of CL linked to a marked linear increase in plasma P4 levels that positively affects the luteal vascularity (LV) (24). Another study has reported a higher average timed velocity (TAV cm/sec) of the luteal artery in pregnant buffalos compared to non-pregnant buffalos (25). Moreover, lower pregnancy rates are observed with lower CL vascularization after 5 days of mating (26). The luteal and early normal pregnancy phases are associated with marked changes in the cardiovascular hemodynamic system, such as elevated blood flow volume and decreased Doppler indices, especially vascular resistance index (27, 28), as all these changes are followed by increased levels of nitric oxide (NO) and its metabolites (NOMs) in the form of nitrite and nitrate, which lead to the improved response of smooth muscle on NO reaction (29). Little is known about the histological structure of CL, using the Doppler technique that is based on the anatomical determination of the luteal artery, as well as characterization of each luteal stage in normal luteal and pregnant phases in Egyptian domestic buffalo. Therefore, this current study aimed to compare the complete growth and development of CL in domestic buffalos from day 5 until day 40 after ovulation either in pregnant or non-pregnant buffalo, and whether luteal vascularity (LV) with progesterone (P4) and nitric oxide (NO) could determine luteal functionality or not.

## Materials and methods

### Ethical approval

All experiments were performed according to the Veterinary Animal Care and Use Committee of the Faculty of Veterinary Medicine, Cairo University (Approval number Vet CU12/10/2021/363).

### Animal housing and management

For the study, cyclic pluriparous (*n* = 18) Egyptian domestic buffalos (*Bubalus bubalis* aged 8–11 years, 3.5 ± 0.5 body condition score, 490 ± 30 kg) kept on a large animal farm in the Faculty of Veterinary Medicine at Giza square (30.0276°N, 31.2101°E) were used. Animals were maintained in open yards. All females were fed a mixed ration that consisted of 60% forage and 40% concentrate, containing dry matter and crude protein. To determine the female cyclicity, all buffalos underwent weekly ultrasound examination (1 time/week for 3 successive weeks) to evaluate the ovarian functionality using EXAGO, rectal ultrasound device (France), as the device is equipped with a 6–12 MHz transrectal probe, before the start of examination procedures.

### Time of synchronization and mating process

Synchronization was performed using the gonadotropin–prostaglandin–gonadotropin combination (GPG/ Ovsynch) protocol, which was previously conducted in cattle (30), and used in buffalo (31). The GPG program was started with the first intramuscular injection of GnRH (5 ml/animal; Receptal^®^ 0.004 mg/ml, Msd Animal Health India) on day 0 followed by a single intramuscular injection of prostaglandin (PGF_2_α) per intramuscular injection (25 mg; Lutalyse^®^, Upjohn) on day 7 and a second GnRH was administered on day 9 as previously demonstrated in buffalos (31). All females underwent routine ultrasound assessment on day 11 after mating from the start of the GPG program and only those excited by a preovulatory follicle ≥1 cm (32) were used in the study (*n* = 18). All buffalos were examined randomly (*n* = 18); after that, nine buffalos were mated naturally 21 h after the second GnRH injection by an adult healthy bull aged from 9 to 10 years (*n* = 5) (33, 34). Mated buffalos were examined on day 25 for pregnancy after natural mating using the same ultrasound device at this day. Of nine females, only six became pregnant (GP I, Pregnant; *n* = 6), while the other three did not (*n* = 3); the second group were not mated and entered in the normal luteal phase after the ovulation process (GP II, Non-pregnant; *n* = 9). Furthermore, ultrasound assessments were done one-day post-mating to assure that all the animals underwent normal ovulation, which was confirmed by the disappearance of the largest follicle using B-mode ultrasound scanning (35, 36).

### CL vascularization assessment

CL ultrasound assessment was conducted every day from day 5 after ovulation until day 40 using Doppler ultrasound portable device (ExaGO, rectal ultrasound device, France) performed with a 6–12 MHz transrectal probe with a device set as follows: velocity was automated at 25 cm/s, Doppler filter was 150 Hz, PRF was 3,500 kHz, and insonation angle was 40° (37). The procedure of CL examination was done once the ovary appeared on the ultrasound scanner and the image was frozen to measure the CL dimensions to estimate the CL area by equation [area = π^*^(a/2)^*^(b/2)], where a and b were the short and long axis of CL dimensions, respectively (26). To evaluate the CL blood flow perfusion, color Doppler mode was activated, and a sample window was placed on the CL tissue to show the colored area in blue (away from the probe) and red (toward probe) color maps, which were assessed by image analysis software program at different stages of the luteal phase and the first days of pregnancy (38).

### CL Doppler parameter evaluations

**Figure 1:**
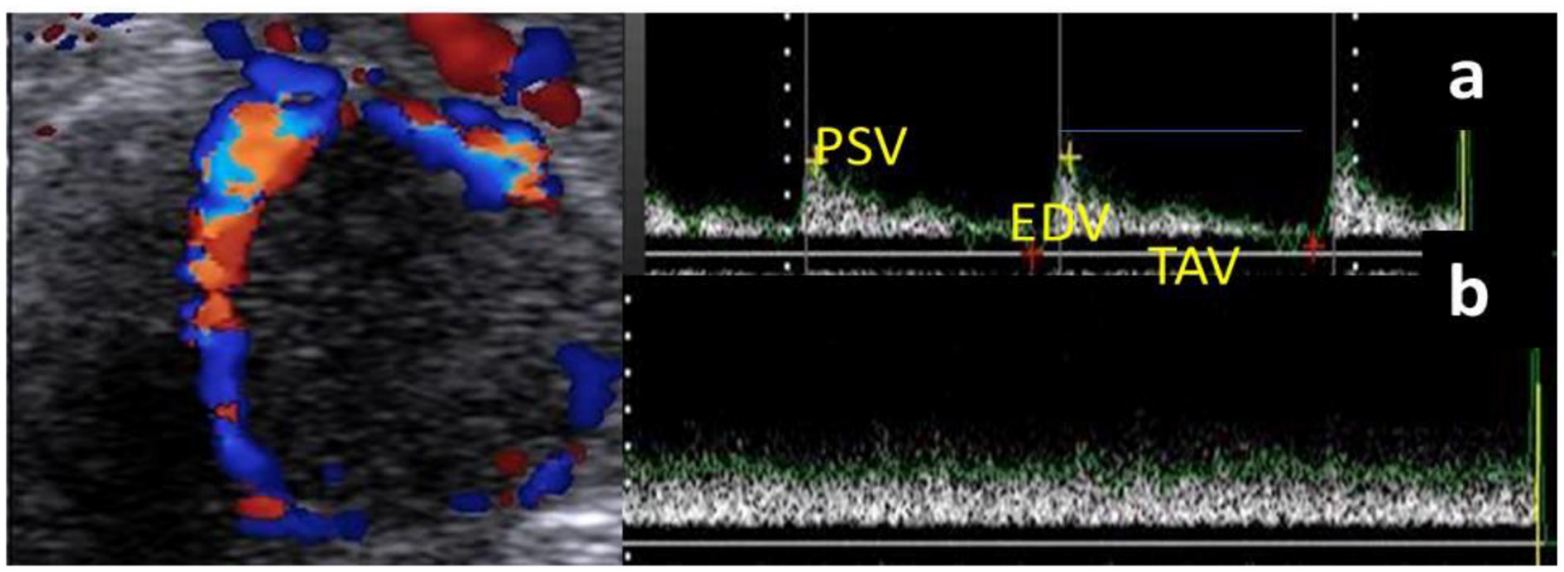
Ultrasonogram revealed the mature corpus luteum (CL) with its spectral wave of both luteal artery **(a)** and luteal vein **(b)** blood flow velocities. N.B: Luteal artery waveform characterized by a complete cardiac cycle in from of peak systolic (PSV; cm/s) and end diastolic velocities (EDV; cm/s) with time average to make maximum velocity (TAV; cm/s), while the vein wave was characterized by absence of cardiac cycle without any spectral graph.

Based on our anatomical determination of the luteal artery and at the level of clearly visualized CL, the spectral mode was activated to show the blood flow velocity of the luteal artery by the wave pattern that was presented only in the luteal artery, not on the luteal vein, as venous circulation assessment did not give any information due to the absence of spectral graph obtained from pulsed-wave Doppler mode (Figure 1). The spectral graph showed a Doppler measurements calculation automatically as resistance and pulsatility index (RI and PI), peak velocity point of contraction and end-diastolic point of relaxation (PSV and EDV cm/s), as well as time to perform maximum velocity (TAV cm/s; Figure 1). B-, color, and spectral modes videos (15 s duration /each) were saved and stored on the flash memory. If there was a cavity in the CL, the area of the CL was assessed by subtracting the cavity area from the whole CL area (39).

### Blood sampling and hormonal assessment

Blood samples were obtained from the jugular vein of all buffalos from day 5 until day 40 following each Doppler examination. Plasma and serum samples were stored at −20 until hormone analysis. Progesterone (P4, EIA-1561) was analyzed using ELISA kits (DRG, Germany) by competitive assaying with inter and intra assays precisions of 9.96 and 5.4, respectively, and test sensitivity of 0.045 ng/ml. Serum samples were used in assaying the nitric oxide (NO) *via* its metabolites (NOMs; μmol/L) as previously examined in our laboratory (40, 41). The nitric oxide inter and intra assay coefficients were 1.17% and 1.09%.

### Collection of tissue samples

**Figure 2:**
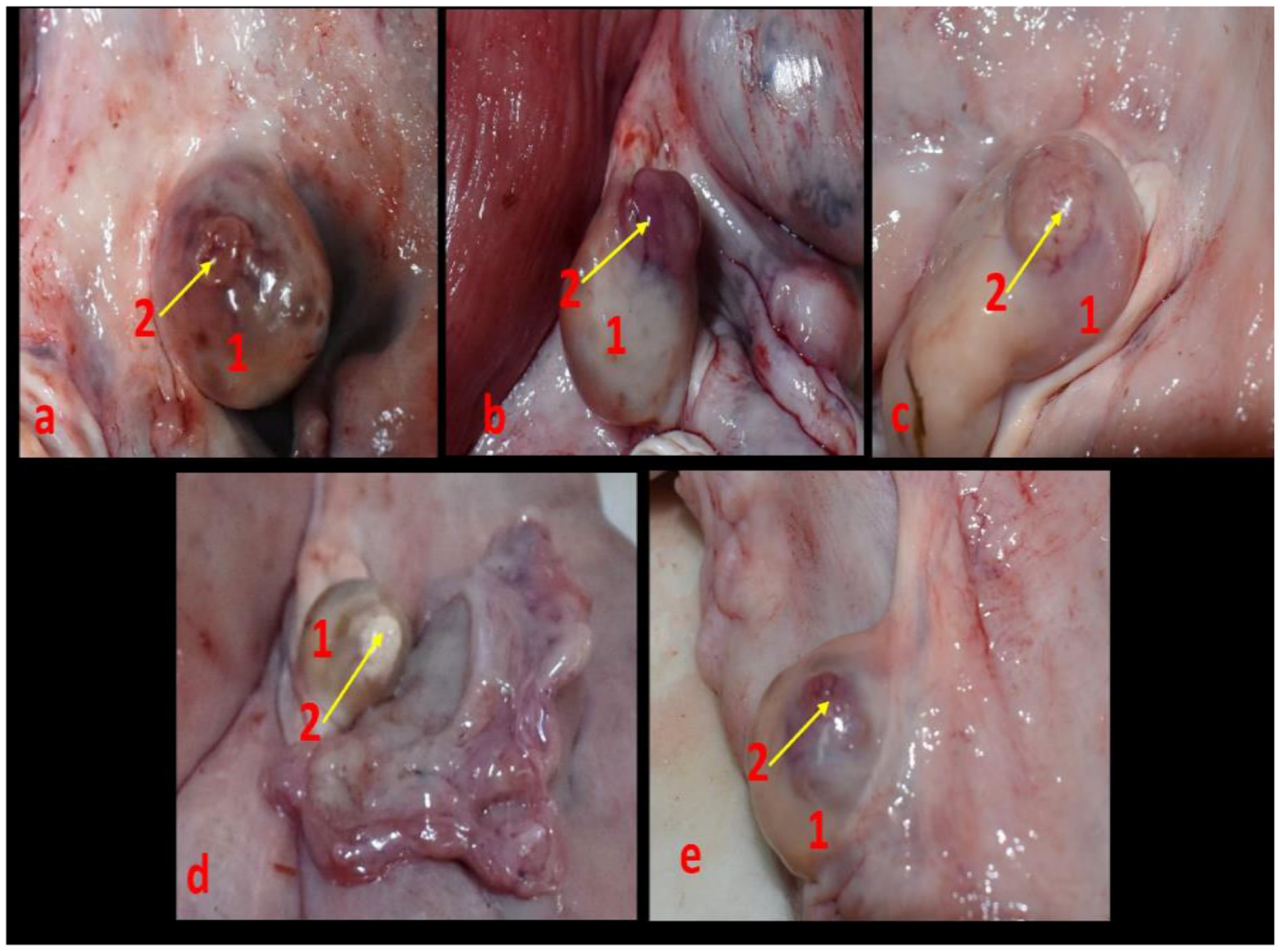
Corpus luteum in the buffalo ovary at different luteal stages as **(a)** represented an early luteal (stage I, 1-5 days), **(b)** demonstrated a mid-luteal (stage II, 6-10 days), **(c)** showed a mid-luteal (stage III, 11–16 days), **(d)** showed a late luteal (stage IV, 17-20 days), and **(e)** demonstrated corpus luteum gravidities at early stage of pregnancy. N.B: 1, ovary and 2, corpus luteum.

Non-pregnant buffalo genitalia (ovaries bearing the CL) (*n* = 18) (*n* = 2 for vascular anatomical architecture and *n* = 16 for histological examination (*n* = 4 genitalia/each luteal stage) in addition to early pregnant buffalo genitalia (*n* = 3) were collected from great Cairo abattoirs within 3 months. The reproductive organs were transported on ice to the laboratory for examination within 15 min after exsanguinations. Stages of the estrous cycle were determined by macroscopic ovarian dating (color, consistency, size, vasculature of CL, and presence of follicles on the surface of the ovary) and then classified into early luteal [Stage I, (*n* = 4), 1–5 days] (Figure 2a) in which the CL appeared small, reddish, and soft; mid-luteal (stage II and stage III) in which the CL became large, brownish, and harder [Stage II, (*n* = 4), 6–10 days] (Figure 2b) and appeared fibrous, pale, and hard [Stage III, (*n* = 4), 11–16 days] (Figure 2c); and late luteal stage [Stage IV, (*n* = 4), 17–20 days] (Figure 2d). CL can be seen as fibrous, yellowish, and hard. While in pregnancy, CL became light reddish with enlargement of the uterine horn (Figure 2e). Additionally, this classification was also defined depending on Baithalu et al. (14).

#### Vascular anatomical architecture

**Figure 3:**
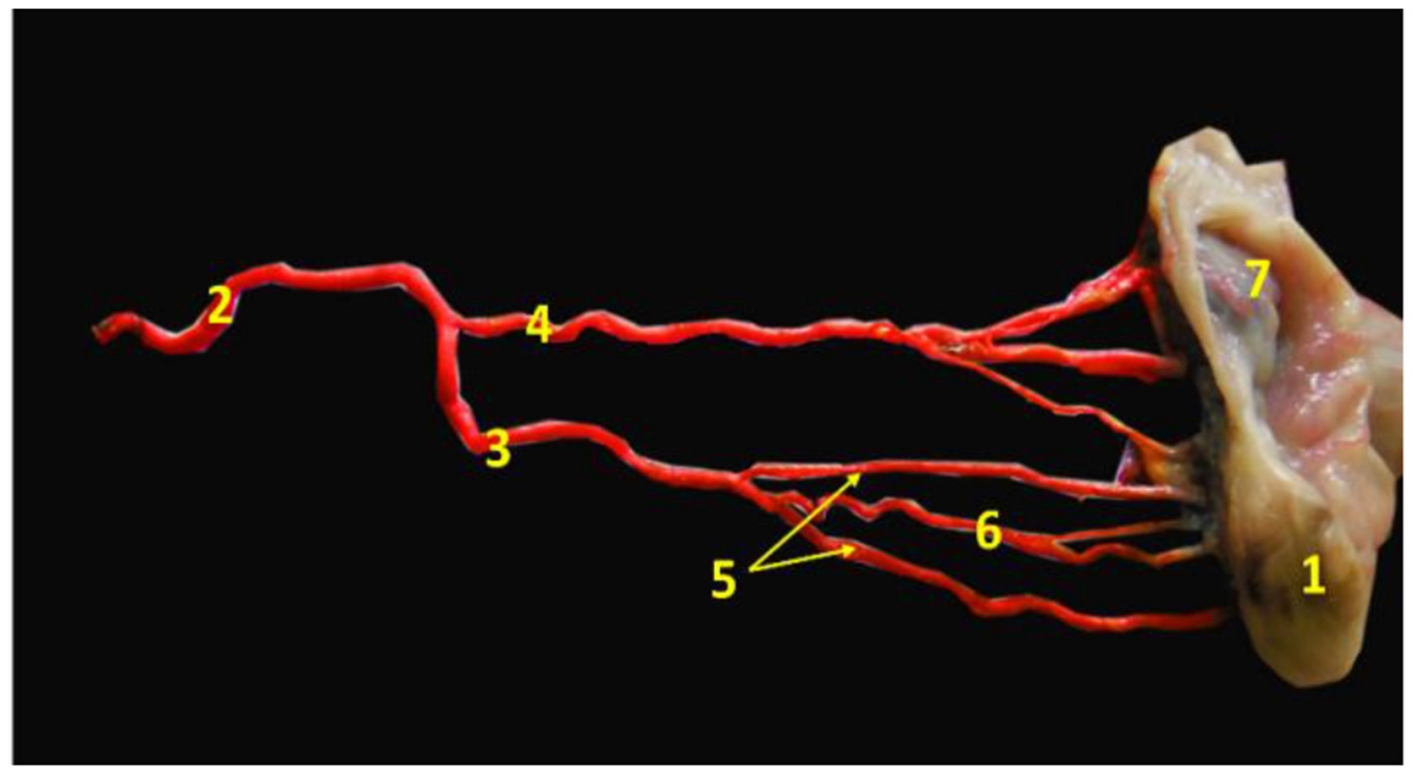
Image revealed the main ovarian artery of the ovary. 1- ovary, 2- main ovarian artery, 3- ovarian branch of the main ovarian artery, 4- tubal branch of the main ovarian artery, 5- ovarian twigs of the ovarian branch, 6- luteal branch of the ovarian branch, and 7- mesoslphenix.

Two specimens from non-pregnant genitalia were used to examine the vascular anatomical architecture for demonstrating the ovarian and luteal artery. Vessels were cannulated, flushed thoroughly with normal saline to remove any blood clots, and then injected with 60% gum milk latex emulsion colored red using ROTRING ink (42, 43). Then the specimens were kept in formalin 10% and 1% glycerine solution for 4 days before manual dissection. The photograph was taken by a digital camera and manipulated by Photoshop ccx64 version. After dissection, we found that the arterial supply of the buffalo ovary was the main ovarian artery, which trifurcated into ovarian, tubal, and uterine branches. The first one continued for a short distance craniolaterally and then divided into 3–4 twigs to enter the ovary from its attached border, the middle one was the luteal artery which was convoluted in its pathway until reached the ovary within the meso-ovarian ligament (Figure 3).

#### Histological examination

Samples of ovarian tissue containing CL from pregnant and non-pregnant buffalos were fixed in 10% neutral buffered formalin, dehydrated in ascending grades of ethanol, cleared in xylene, and finally embedded in paraffin wax. Paraffin sections (4–5 μm thick) were obtained and stained with hematoxylin and eosin, Crossman's trichrome stain, and periodic acid-Schiff (PAS) (44).

### Statistical analysis

All data are presented as the mean ± SEM as all results are the first check for normality. An unpaired *t*-test was used for comparisons between the two groups at each time point. The statistical significance of progesterone and nitric oxide alterations, as well as luteal Doppler findings in both groups, was assessed by repeated-measures two-way analysis of variance (ANOVA) to study the effect of group, time, and their interaction. By this method, you can compare all 16 values (2^*^8) (interaction between the effect of group and time). All analyses were achieved by using SPSS software version 20. *p* < 0.05 indicates significant differences. Pearson's correlation coefficients between progesterone levels and CL Doppler parameters in all females (pregnant and non-pregnant) were calculated.

## Results

### Corpus luteum characterization in live animals and hormonal analysis

**Figure 4:**
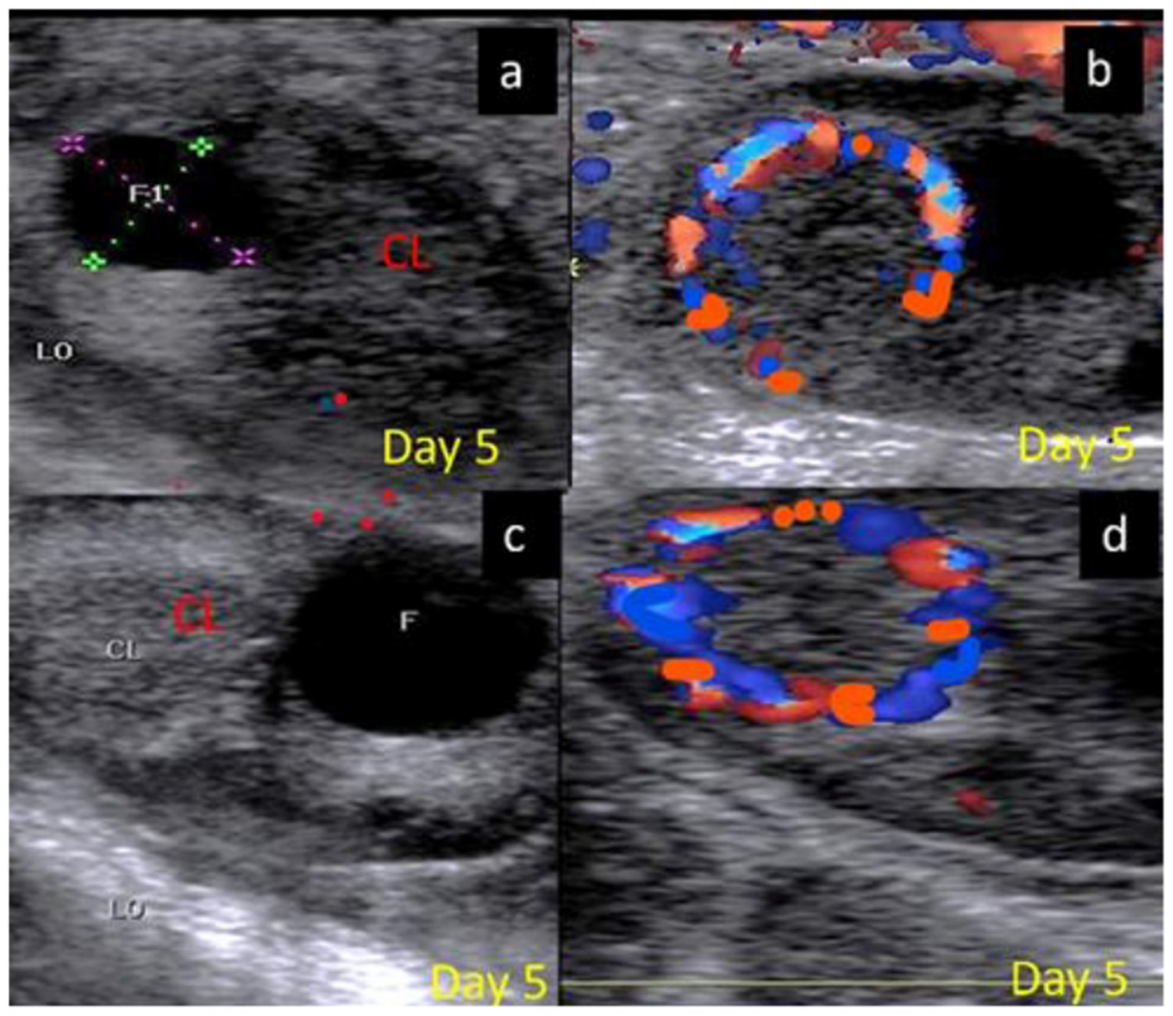
B-mode and colored ultrasonograms of a 9-year-old buffalos at the early luteal phase (stage I; from 1 to 5 days) as **(A,B)** images showed an early formed corpus luteum in non-pregnant female on day 5 in gray and color modes and **(C,D)** images showed an early formed corpus luteum in suspected pregnant one on day 5 in gray and color modes. CL, corpus luteum.

**Figure 5:**
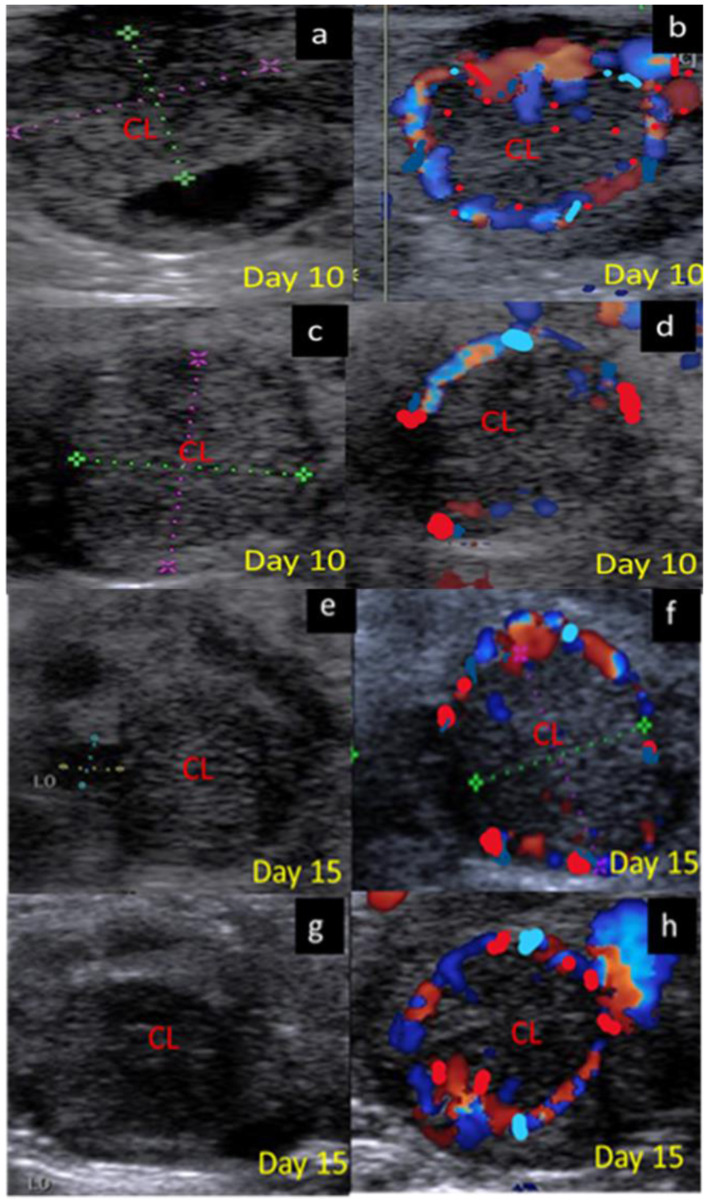
B-mode and colored ultrasonograms of a 9-year-old buffalos at the mid luteal phase with two stages stage II; from 6 to 10 days and stage; III from 11 to 16 days, as **(a,b)** images showed the corpus luteum in non-pregnant female on day 10 in gray and color modes, **(c,d)** images showed the formed corpus luteum in suspected pregnant one on day 10 in gray and color modes, **(e,f)** images showed the corpus luteum in non-pregnant female on day 15 in gray and color modes, and **(g,h)** images showed the formed corpus luteum in suspected pregnant one on day 15 in gray and color modes. CL, corpus luteum.

**Figure 6:**
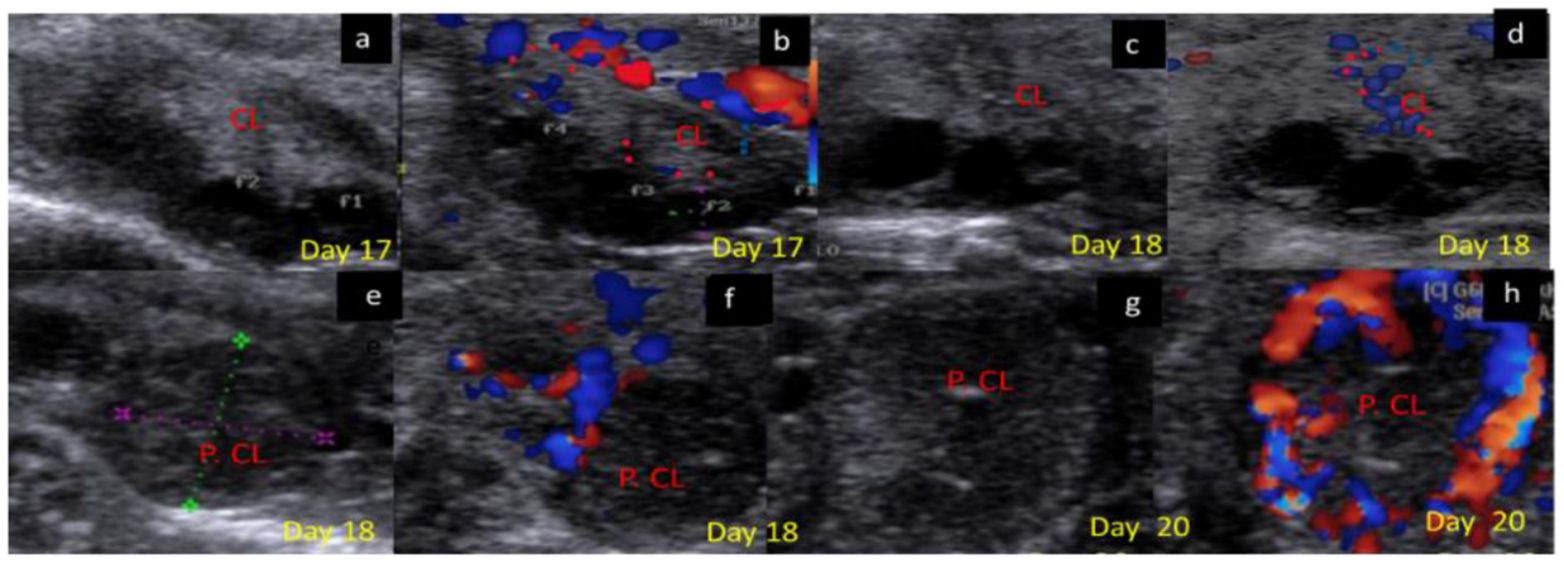
B-mode and colored ultrasonograms of 9-year-old buffalos at the late luteal in non-pregnant compared to pregnant one (Stage IV vs. Stage I suspected pregnant from 17 to 20 days). As **(a,b)** images showed the regressed corpus luteum on day 17 in gray and color modes and **(c,d)** images showed the more regressed corpus luteum on day 18 in gray and color modes compared to **(e,f)** images showed the corpus luteum in suspected pregnant females on day 18 in gray and color modes, and **(g,h)** images showed the corpus luteum in suspected pregnant females on day 20 in gray and color modes. CL, corpus luteum.

The luteal phase was divided into early (Figure 4), mid [stages II and III] (Figure 5), and late (Figure 6) stages in pregnant and non-pregnant animals. Moreover, this luteal classification was also confirmed by the progesterone levels at these stages.

#### CL area (cm^2^) determination by B-mode ultrasonography

**Figure 7:**
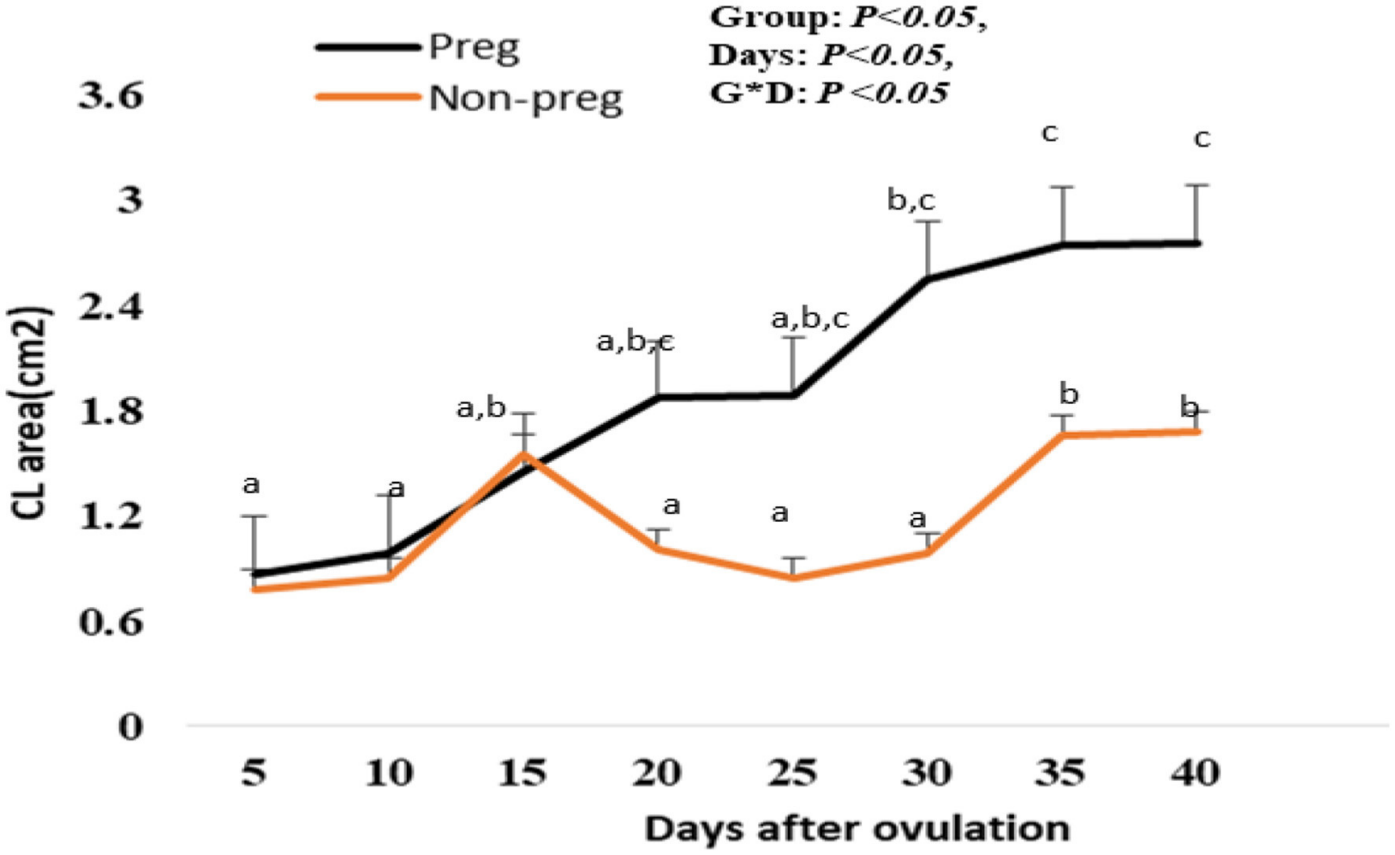
Area of the corpus luteum (CL area; cm^2^) presented in pregnant (Preg) and non-pregnant (Non-preg) buffalos from day 5 till day 40 after ovulation. Data are obtained as mean with standard error of mean. ^a, b^ Values are significantly different at *P* < 0.05 compared with day 5 in both groups, while ^c^value is significantly different at *P* < 0.05 between two groups at the indicated same time point.

The CL area (cm^2^) in both groups showed an elevation beginning from day 5, and with each examination time point, there was an additional increase till day 15 after ovulation. Also, the CL area in non-pregnant buffalos showed a similar pattern but a non-significant decline was observed on days 20, 25, and 30, then the CL area was significantly (*p* < 0.05) elevated on days 35 and 40. The differences between the pregnant and non-pregnant groups reach a significant (*p* < 0.05) level from day 20 (1.87 ± 0.02 cm^2^) until day 40 (2.75 ± 1.85 cm^2^). The time and interaction between the time with the group showed a significant (*p* < 0.05) difference in the CL area (Figure 7).

#### Luteal artery Doppler parameter evaluations

**Figure 8:**
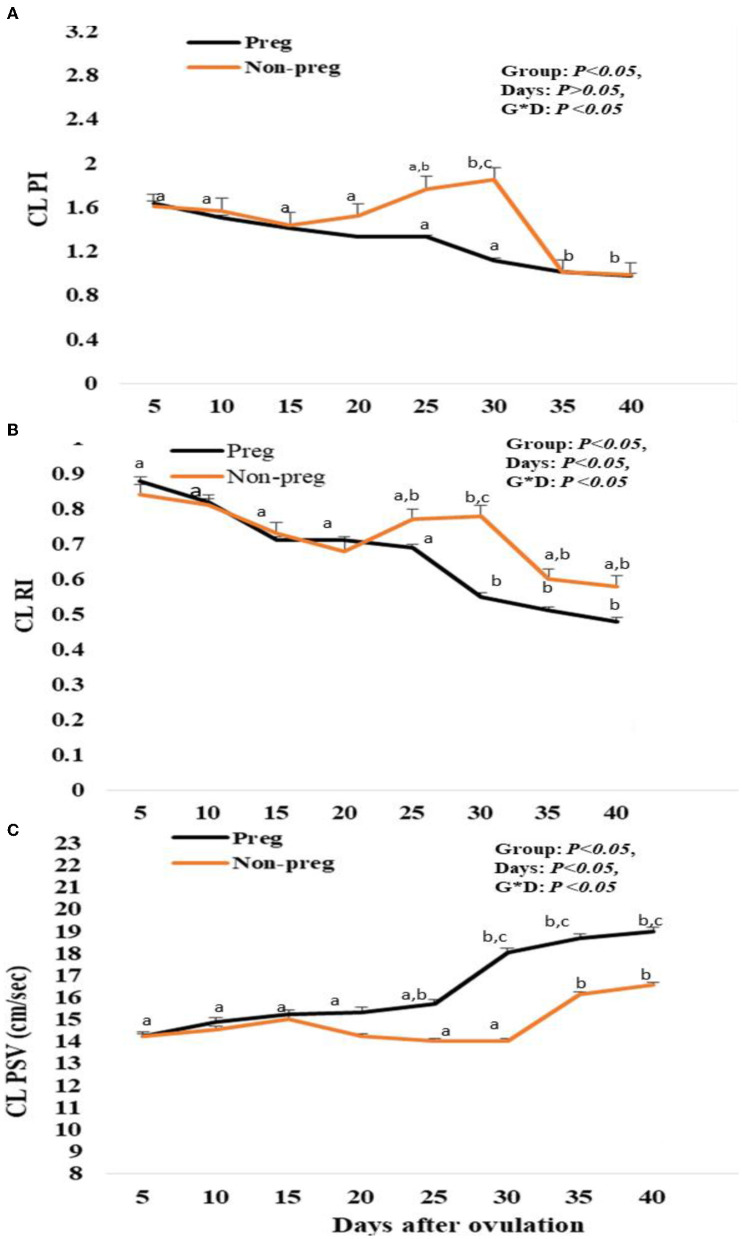
Pulsatility index (PI; **A**), resistance index (RI; **B**) and luteal peak systolic velocity (PSV; cm/sec; **C**) values presented in pregnant (Preg) and non-pregnant (Non-preg) buffalos from day 5 till day 40 after ovulation. Data are obtained as mean with standard error of mean. ^a, b^ Values are significantly different at *P* < 0.05 compared with day 5 in both groups, while ^c^value is significantly different at *P* < 0.05 between two groups at the indicated same time point.

The luteal artery was the second division of the ovarian branch of the main ovarian artery and lodged within the meso-ovarian ligament (Figure 3). The spectral mode of the luteal artery was measured to determine both luteal Doppler indices that were expressed by RI and PI. Luteal PI in both groups showed a decrease beginning from day 5, and with each examination time point, there was an additional decrease till day 15. Also, the PI in non-pregnant buffalos showed a similar pattern with a maximum elevation on day 30. The differences between the pregnant and non-pregnant groups reach a significant (*p* < 0.05) level on day 20 (1.33 ± 0.01) and continue to be significant till day 40 (0.89 ± 0.01; Figure 8A). The interaction between time with the group had shown a significant (*p* < 0.05) difference in luteal PI, while the time did not show any significant difference.

Luteal RI in both groups showed a decrease beginning from day 5, and with each examination time point, there was an additional decrease. Also, in non-pregnant buffalos, the luteal RI showed a similar pattern with a maximum elevation on day 30. The differences between the pregnant and non-pregnant groups reach a significant (*p* < 0.05) reduction from day 25 (0.69 ± 0.01) until day 40 (0.48 ± 0.01) in the pregnant group compared to that in the non-pregnant group (Figure 8B). The time and interaction between the time with the group show a significant (*p* < 0.05) difference in luteal RI.

Finally, the spectral luteal PSV (cm/s) was elevated in both groups, but the elevation was significant (*p* < 0.05) from day 25 (15.88 ± 0.33) to day 40 (18.97 ± 0.74) in the pregnant group, while in the non-pregnant group, the PSV was elevated until day 15 (15.05 ± 0.85) after ovulation and then subsequently declined at days 20, 25, and 30, and then elevated at day 35 and day 40, as the time and interaction between time with the group showed a significant (*p* < 0.05) difference in luteal PSV (Figure 8C).

#### Progesterone and nitric oxide levels

**Figure 9:**
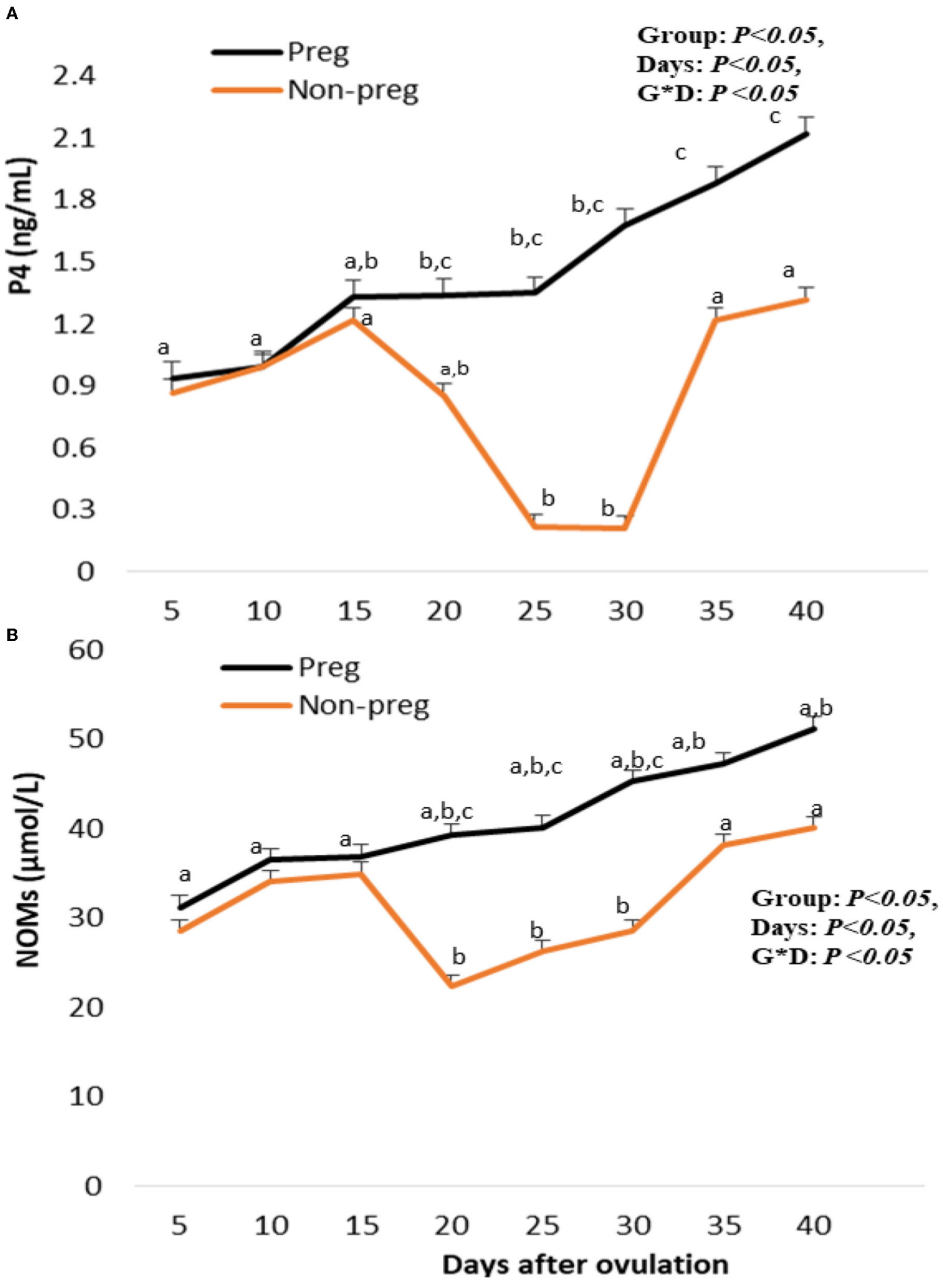
Plasma progesterone (P4; ng/mL; **A**), and serum nitric oxdie metabolites (NOMs; μmol/L; **B**) levels presented in pregnant (Preg) and non-pregnant (Non-preg) buffalos from day 5 till day 40 after ovulation. Data are obtained as mean with standard error of mean. ^a, b^ Values are significantly different at *P* < 0.05 compared with day 5 in both groups, while ^c^ value is significantly different at *P* < 0.05 between two groups at the indicated same time point.

Plasma progesterone levels in both groups showed an increase beginning from day 5, and with each examination time point, there was an additional increase. Plasma progesterone levels in pregnant females were elevated (*p* < 0.05) from day 20 (1.34 ± 0.01) until day 40 (2.21 ± 0.01), while those in non-pregnant females significantly decreased at days 25 and 30 (0.23 ± 0.01 and 0.21 ± 0.02) (Figure 9A). The time and interaction between time with the group showed a significant (*p* < 0.05) difference in progesterone level. Serum NOM levels in both groups increased from day 5 till day 15. In the pregnant group, NOM levels were elevated significantly (*p* < 0.05) from day 20 to day 40 compared to the non-pregnant group (Figure 9B), in addition, the time and interaction between time with both groups showed a significant (*p* < 0.05) difference in NOM. A positive correlation (*p* < 0.01) was observed for both pregnant and non-pregnant buffalos between plasma levels of P4 and CL PSV (cm/s), while there was a negative correlation (*p* < 0.01) between P4 levels and both Doppler indices (RI and PI).

### Uterine blood flow in live animals

**Figure 10:**
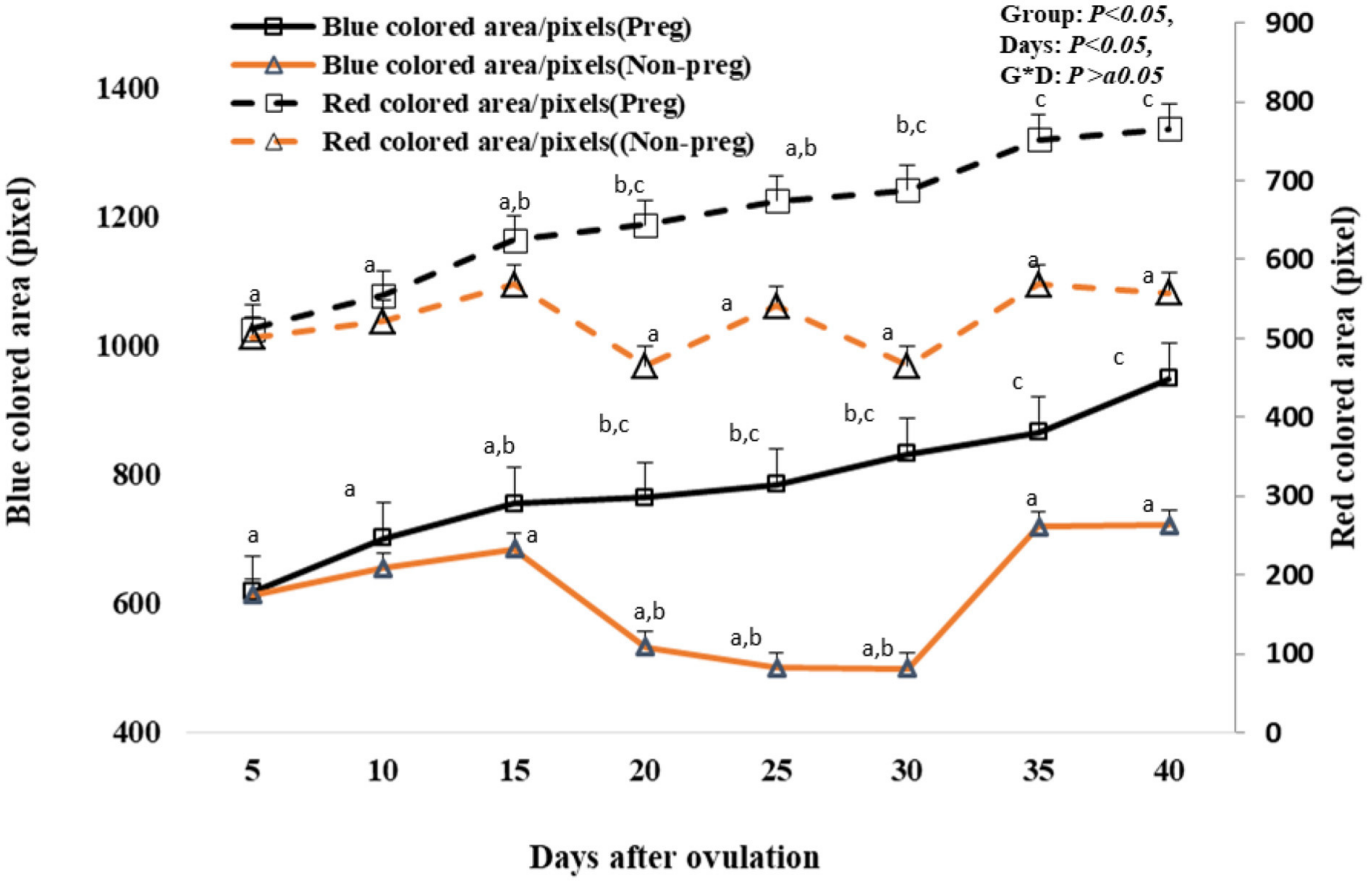
Uterine blood flow area expressed by blue and red colored area/pixels presented in pregnant (Preg) and non-pregnant (Non-preg) buffalos from day 5 till day 40 after ovulation. Data are obtained as mean with standard error of mean. ^a, b^ Values are significantly different at *P* < 0.05 compared with day 5 in both groups, while ^c^ value is significantly different at *P* < 0.05 between two groups at the indicated same time point.

Both blue and red colored area/pixels were seen to be significant (*p* < 0.05) elevated from day 20 (764.12 ± 4.21 and 644.21 ± 2.51) till day 40 (948.12 ± 12.42 and 766.32 ± 3.55) in pregnant females compared to non-pregnant females as shown in Figure 10. There was no time and group interaction in the red and blue uterine blood flow.

### Histological investigation

**Figure 11:**
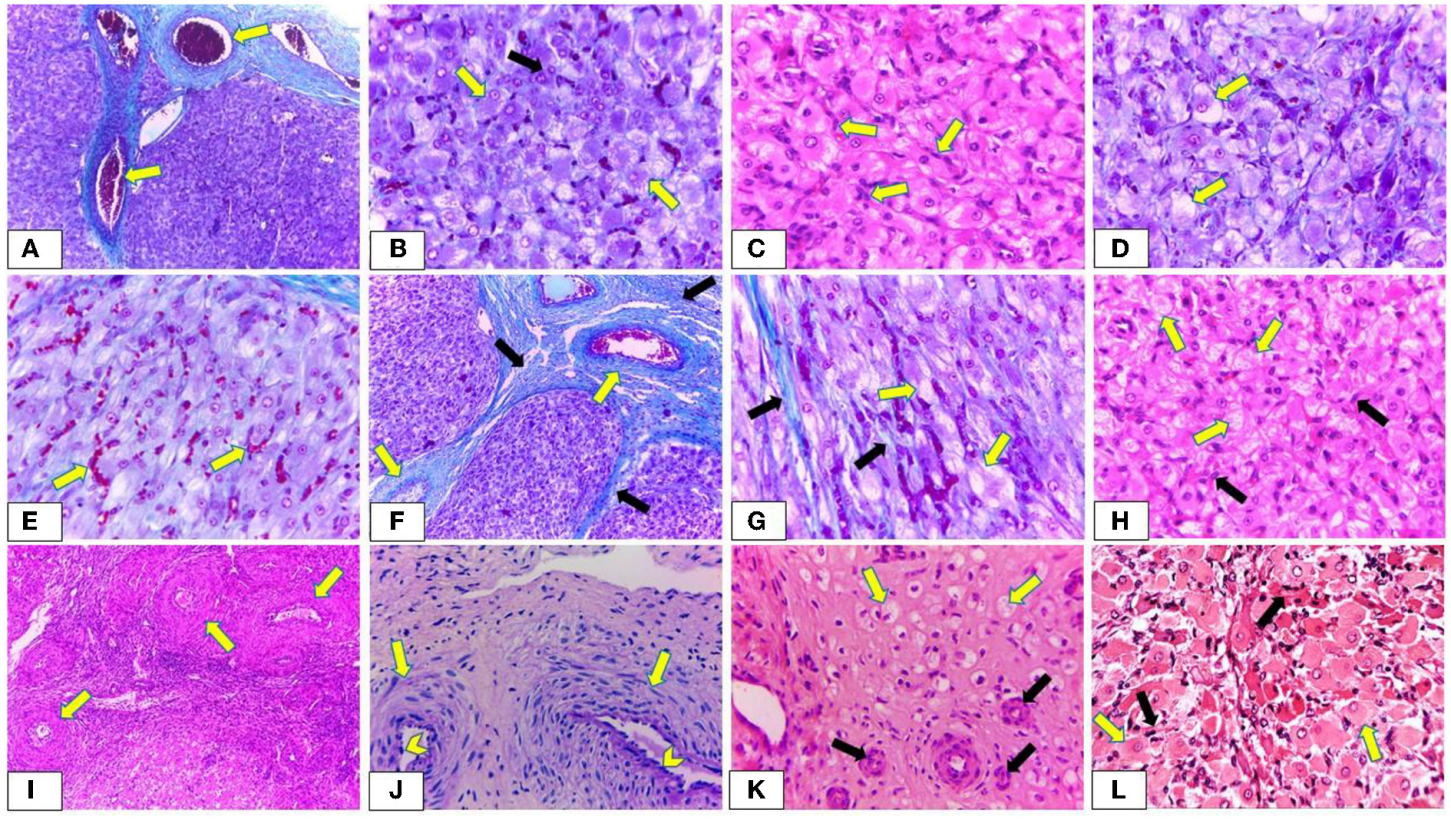
Photomicrograph showing the histological structure of corpus luteum (CL) during oestrus cycle and pregnancy. **(A,B)** Demonstrating early luteal stage (Stage I). **(A)** The CL is covered by a highly vascularized connective tissue capsule and septa. The blood vessels are dilated and engorged with blood (arrow) (Crossman's trichrome stain, x100). **(B)** Large lutein cells (LLCs) are ovoid, or polyhedral in shape with large spherical vesicular eccentrically situated nuclei and prominent nucleoli (yellow arrow) while small lutein cells are irregular in shape with eccentrical spherical lightly stained nuclei (SLCs) (black arrow). Furthermore, LLCs possessed more cytoplasmic: nuclear ratio and lipid droplets than that of SLCs (Crossman's trichrome stain, x400). **(C,E)** Showing mid luteal stage (stage II). **(C)** Presence of numerous fibroblast cells (arrow) with increasing the size of LLCs (H & E, x400). **(D)** Revealing a high number of vacuolated lutein cells with large vacuoles (arrow) (Crossman's trichrome stain, x400). **(E)** Most lutein cells appear in a close association with engorged blood capillaries (arrow) (Crossman's trichrome stain, x400). **(F,H)** Revealing mid luteal stage (stage III). **(F)** Presence of a high amount of collagen fibers in the capsule surrounding CL and C.T. septae (black arrow) in addition to increasing the thickness of the blood vessels enclosed in both capsule and septae (yellow arrow) (Crossman's trichrome stain, x100). **(G)** Presence of a high amount of collagen fibers in the interstitial tissue (black arrow). Furthermore, LLCs appear with abundant large vacuoles which are represented by narrow strands in some cells (yellow arrow) (Crossman's trichrome stain, x400). **(H)** LLCs (yellow arrow) appear with abundant large vacuoles distributed among numerous fibroblast cells (black arrow) (H & E, x400). **(I,K)** Exhibiting late luteal stage (stage IV). **(I)** A substantial increase in the thickening of blood vessels enclosed within the C.T. capsule (arrow) (H & E, x400). **(J)** Showing regressed capillaries and arterioles like remnants with an onion-skin arrangement of surrounding myofibroblast (arrow) and heavily condensed and rounded endothelial cells (chevron) (PAS, x400). **(K)** Most lutein cells appear shrunken, degenerated, highly vacuolated with small, condensed spherical or oval peripherally situated nuclei (yellow arrow). Moreover, presence of regressed blood vessels (black arrow) (H & E, x400). **(L)** Showing CL of pregnant buffalos has the same structure of CL at stages I and II with abundant LLCs (yellow arrow) in close association with blood capillaries (black arrow) (H & E, x400). The yellow arrows indicate the prominent nuclei with some LLCs.

CL of non-pregnant buffalos demonstrated different stages of activity. In the early luteal stage (Stage I), the CL was covered by a fibrous connective tissue capsule, which formed septae that divided the CL into lobules. In this stage, both the capsule and septae appeared highly vascularized with many dilated and engorged blood vessels (Figure 11A). Some lutein cells were large, ovoid, or polyhedral in shape with large spherical vesicular eccentrically situated nuclei and prominent nucleoli [large lutein cells (LLCs)], while others were small and irregular in shape with eccentrically spherical lightly stained nuclei [small granulosa lutein cells (SLCs)]. LLCs possessed more cytoplasmic: nuclear ratio and lipid droplets than that of SLCs. The LLCs occupied a more central portion of CL in a close association with blood capillaries, while SLCs occupied the periphery portion and were distributed among the large ones (Figure 11B). The mid-luteal stage (stage II) was similar to stage I, except for the presence of numerous fibroblast cells and a high number of vacuolated cells with large vacuoles and an increasing number of lutein cells (Figures 11C,D). Moreover, most cells appeared in close association with engorged blood capillaries (Figure 11E). The mid-luteal (Stage III) was characterized by the presence of a high amount of collagen fibers in the capsule surrounding CL, septae, and interstitial tissue in addition to increasing the thickness of the blood vessels enclosed in both capsule and septae (Figures 11F,G). Furthermore, some luteal cells appeared to be generated with shrunken condensed nuclei, while others showed the apocrine mode of secretion. In this mode of secretion, the remaining part of the cells appeared with deep acidophilic cytoplasm and condensed nucleus. Moreover, LLCs appeared with abundant large vacuoles distributed among numerous fibroblast cells. These vacuoles were represented by narrow strands in some cells (Figures 11G,H). Conversely, the late luteal stage (Stage IV) was characterized by a substantial increase in the amount of fibrous CT mainly collagen, thickening of the CT capsule, CT septae, and blood vessels (Figure 11I) in addition to regressed capillaries and arterioles like remnants with an onion-skin arrangement of surrounding myofibroblast and heavily condensed and rounded endothelial cells (Figure 11J). Furthermore, most luteal cells appeared shrunken, degenerated, and highly vacuolated with small, condensed spherical or oval peripherally situated nuclei (Figure 11K). Additionally, the CL of pregnant buffalos revealed the same structure of CL at stage II with abundant LLCs surrounded by highly vascularized connective tissue capsules (Figure 11L).

## Discussion

The current study compares the complete growth and development of CL in domestic buffalos from day 5 until day 40 after ovulation. CL plays a critical role in the gestation maintenance and establishment in many species because of its ability to produce an adequate P4 level (10, 45), therefore, any decline of this mechanism could affect embryonic mortality as previously demonstrated in cows (46). This study shows a significant elevation in CL area from day 20 until day 40 in pregnant buffalos compared to non-pregnant buffalos with a marked elevation of plasma P4 levels in the same group, which could help determine the importance of adequate CL diameter and area to establish the first early stage of pregnancy (47). Additionally, some studies reported a higher CL area in early pregnant females compared to non-pregnant ones (25), but others did not show a significant difference between the two groups (48). Similarly, some studies reported an elevation in plasma P4 levels from day 7 to day 10 after mating in pregnant females (25, 47) as high P4 levels were responsible mainly for greater embryonic development (23). In addition, the P4 levels remained elevated in some studies until 6–8 months of pregnancy (49).

Increases in CL diameter and progesterone level were greater in pregnant than in non-pregnant buffalos, indicating the importance of adequate CL size for maintenance of pregnancy (47). Lesser plasma progesterone levels from day 10 to 20 after AI in buffalos were associated with the death of an embryo (22), demonstrating the pivotal role of CL for pregnancy stage development and maintenance. The variation in the serum P4 concentrations throughout the estrous cycle of non-pregnant and pregnant buffalos is primarily dependent on the blood flow, CL area, amount of steroidogenic tissue (number and size of luteal cells), and its capacity to synthesize progesterone (10), in addition to its lipid contents (50). The development, maintenance, and regression of CL involved remarkable morphological and functional changes during the estrous cycle (51–53). The current study showed that CL had steroidogenic cells involving LLCs and SLCs with morphological characteristics that were reported by Baithalu et al. (14) in buffalo and Ozen et al. (54) and Xavier et al. (55) in a cow. The presence of large lightly stained nuclei of LLCs throughout stages I, II, and III may indicate the activity and hypersecretory LLCs during these phases of the cycle and pregnancy, while luteal cells of Stage IV revealed small more dense nuclei, which is an indication of luteolysis. Furthermore, the presence of vacuolated cytoplasm of LLCs was caused by their lipid contents that vary throughout the estrous cycle and appear in a close association with the synthesis and secretion of progesterone. Kapoor et al. (50) demonstrated that the variations in lipid distribution within the cyclic and regressed CL were inversely associated with the activity of the 3β-hydroxysteroid dehydrogenase enzyme involved in the synthesis of steroid hormones. Our results exhibited lutein cells of stage IV with highly vacuolated cytoplasm. Increasing accumulation of lipids in the regressed luteal cells with a significant decline in production of P4 hormone might be due to degenerated smooth endoplasmic reticulum resulting in absence of 3β-HSD in addition to degenerated mitochondria containing P450 side-chain cleavage (50, 56). Both 3β-HSD and P450 side-chain cleavage were involved in the biosynthesis of progesterone (50). Additionally, during the estrus cycle, the luteal cell population and the size of luteal cells increased and then regressed at stage IV. These results were consistent with references (14, 57, 58). Therefore, these findings reflect the variation in the serum P4 concentrations throughout the estrous cycle of non-pregnant and pregnant buffalos.

Hence, the present study was designed to study the cellular composition of the mature buffalo CL with its functional characterization in relation to progesterone secretory ability and nitric oxide during the normal luteal and pregnancy phases. In the normal luteal phase the level of NOMs was critical as it was related to the luteal vascularization and functionality (59), as after ovulation nitric oxide affects oocyte activation by regulating the calcium channel during the process of fertilization (60), while in the normal early pregnant stage, nitric oxide contributes to the elevation of maternal blood and reduction of blood pressure (61). Moreover, nitric oxide was extremely important in the embryo's multiple divisions (62) as some studies showed that embryonic growth was delayed due to the presence of inhibitory mediators in the blastocyst stage that adversely affects nitric oxide levels (63). Besides nitric oxide, some mediators have been concerned with this phenomenon, such as estradiol and prostacyclin (64).

The elevation in uterine blood flow that was expressed by red and blue colored areas in the pregnant group compared to non-pregnant buffalos could be associated with the buffalo maternal pregnancy recognition that began once the early embryo moved in the fallopian tube and then entered the uterus after 5–6 days post-mating, as the uterine vascularization is included in the implantation procedures expressed by the growth and development of new blood vessels (65, 66). Furthermore, increased serum levels of NOMs in early pregnancy were very important in the vasodilator mechanisms as nitric oxide is shared in the blood pressure regulations and coronary artery vasomotion, therefore any abnormalities in nitric oxide and its metabolites levels could adversely affect pregnancy (67) in form of hypertension and angiogenesis problems (68).

Besides progesterone's important function, CL functionality could also be assessed by luteal vascularization (36). In this study, the CL vascularization was determined using both Doppler indices and PSV (cm/s), as both Doppler indices decreased in pregnant females compared to those in non-pregnant females, while the luteal artery PSV was elevated in the same pregnant group, this could be explained by the inverse relationship between both Doppler indices and Doppler velocities with time average point (TAV) as previously reported in other studies (69–72), in addition, an inverse relationship was observed between blood flow rate and Doppler indices especially PI (73–75). In accordance with our finding, a study reported an increase in both peak and time average velocities (PSV and TAV cm/s) on day 7 after time artificial insemination in pregnant cows (76), as the determination of luteal blood flow total area with Doppler velocities could increase the accuracy of pregnancy prediction (26). Consistent with our Doppler measurements, cyclic CL (stage I, II, and III) and CL of pregnancy were characterized by high vascularization, and most lutein cells were adjacent to engorged blood capillaries that were in harmony with Xavier et al. (55) in the pregnant cow. These results might be due to the high metabolic demand for CL. Moreover, the growth and maintenance of CL and its adequate endocrine function were mainly associated with increasing luteal vascularization and angiogenesis (77, 78). Many studies found a similar positive correlation between CL vascularization in its blood flow and plasma P4 levels after ovulation (79). However, during luteal regression, all lutein cells decreased in their number and shrunk until they disappeared, leaving arteriole-like remnants of blood vessels with an onion-skin arrangement of surrounding myofibroblast and heavily condensed and rounded endothelial cells with dense connective tissue in the residual CL. This finding was in accordance with observations reported by Augustin et al. (80). Increasing thickness of the CT capsule, CT septae, and blood vessels led to reduced blood flow, which in turn resulted in decreased progesterone secretion as observed in the current study.

Moreover, this study revealed the presence of non-steroidogenic cells, mainly fibroblast and endothelial cells. In stages III and IV, the number of fibroblasts notably increased, which was consistent with the results of Baithalu et al. (14) as the fibroblasts were responsible for the synthesis of connective tissue fibers and extracellular matrix (81), and collagen fibers were required to alternate the degenerated and regressed luteal cells. Baithalu et al. (14) reported that, during the late luteal stage, the greater number of macrophages and fibroblasts could serve as a cellular marker of luteal regression. Furthermore, our results revealed a high rate of deposition of collagen fibers in stages III and IV which comes in accordance with Jaglan et al. (53), who observed changes in the collagen concentration with the development and regression of cyclic buffalo CL during the estrous cycle. Iwahashi et al. (82) showed that alterations in the synthesis and distribution of collagen played a primary role in determining the CL structure and function.

## Conclusion

The histological structure of CL and assessment of its hemodynamics depending on anatomical identifications could be used extensively to get useful data about CL functional status in both luteal and early pregnant phases. Finally, the evaluation of the luteal artery could be extremely helpful as the artery showed a wave pattern to determine the potential benefits of colored and pulsed Doppler in CL vascularization assessment.

## Data availability statement

The original contributions presented in the study are included in the article/supplementary material, further inquiries can be directed to the corresponding author.

## Ethics statement

The animal study was reviewed and approved by Faculty of Veterinary Medicine, Cairo University.

## Author contributions

EA, IE, YA, AT, NY, and SD designed the protocol and collected the samples. NY worked on the histological examinations. EA and IE performed Doppler and ultrasonographical images, while YA, AT, and SD performed the anatomical vascular architecture. All authors drafted the manuscript, reviewed it, and approved the last version of the manuscript.

## Conflict of interest

The authors declare that the research was conducted in the absence of any commercial or financial relationships that could be construed as a potential conflict of interest.

## Publisher's note

All claims expressed in this article are solely those of the authors and do not necessarily represent those of their affiliated organizations, or those of the publisher, the editors and the reviewers. Any product that may be evaluated in this article, or claim that may be made by its manufacturer, is not guaranteed or endorsed by the publisher.
